# Healthy and sustainable diets from today to 2050—The role of international trade

**DOI:** 10.1371/journal.pone.0264729

**Published:** 2022-05-18

**Authors:** Brendan R. Mapes, Steven D. Prager, Christophe Béné, Carlos Eduardo Gonzalez

**Affiliations:** 1 Food Environment and Consumer Behavior, International Center for Tropical Agriculture, Cali-Palmira, Cali, Colombia; 2 DevTech Systems Inc, Arlington, Virginia, United States of America; 3 Climate Action, International Center for Tropical Agriculture, Cali-Palmira, Cali, Colombia; Universidad Nacional Autonoma de Nicaragua Leon, NICARAGUA

## Abstract

The connection between international trade and food systems (un)sustainability is both contentious and critical for policy work supporting progress towards achieving the twin goals of hunger alleviation and dietary health while improving the overall sustainability of development. We characterize the food system using a set of metrics based upon the EAT-Lancet commission dietary guidelines for both over- and under-consumption of different foods to assess country-level dietary health and sustainability in tandem. Using a partial equilibrium model of agricultural production and trade, we then project the functioning of the global agricultural system to 2050 and calculate the metrics for that year. For most regions we find increased overconsumption above the expert-defined healthy and sustainable diet thresholds, with more limited progress towards closing dietary health and sustainability gaps where they currently exist. Trade influences this dynamic into the future under certain socioeconomic conditions, and we find that under a “business as usual” trade environment, future agricultural import profiles continue to be misaligned with dietary health and sustainability outcomes, suggesting the potential for early intervention in trade policy as a means to positively influence food system outcomes.

## Introduction

The impact of global agricultural trade on the environment and human health is a contested space in agricultural policy. The importance of the relationship between trade and dietary health is well established in the literature, especially as it relates to the question of trade liberalization [1–5]. Supporters of food sovereignty movements argue that globalized agricultural trade deprives communities and countries of the ability to chart their own path towards broader socioeconomic development and sustainability, and question the methods of global food policy and trade institutions in contributing to food price shocks and commodity dumping [6–8]. Others point to the role of globalized food trade in driving increased production and consumption of highly processed foodstuffs [2, 9], localized biodiversity loss [10, 11], and increased waste through an agricultural Jevon’s paradox [12].

On the other side, proponents of global agricultural trade point to reduced hunger and increased opportunities for agricultural producers in the developing world. They highlight increased opportunities for food system actors to buy and sell goods via integration into global value chains and the expansion of robust agricultural markets to more and more communities [13]. The authors in [14] highlight the opportunities associated with the role agricultural trade plays in facilitating adaptation to climate change through leveraging distinct regional specificities (e.g., available natural resources) and specialization. Consistent with [14] who highlight the need for “sensitive implementation” of trade, the authors in [15] suggest that trade may produce food system sustainability benefits for some groups of countries but not others.

Trade is clearly a critical component of the global food economy, but the lack of consensus regarding the specific effects of trade indicates that there is much left to understand about the potential roles of trade in supporting food system sustainability. While there are broad explorations of the relationship between trade and dietary (un)sustainability [16–18] in the food policy literature, we would benefit greatly from a better understanding of how trade can serve as a tool in fostering food system sustainability while at the same time avoiding potentially perverse consequences of trade policy decisions. Food systems are evolving rapidly, and forward-looking analysis is thus useful in anticipating how food systems might respond to the different drivers and structures that shape them. The challenge is immense and must thus be broken into manageable pieces; this research analyzes and expands upon the relationship between trade and one facet of broader food systems sustainability—sustainable and healthy diets—to address the question: “can we trade our way to sustainable and healthy diets?” To answer this question we build upon work leveraging global agricultural foresight models to explore different dietary trajectories [19, 20], and highlighting the importance of trade in global food systems foresight frameworks [21].

To this end, we operationalize two quantitative measures of dietary quality using the recent EAT-Lancet commission on Food, Planet, and Health’s dietary guidelines and food availability data from the FAOSTAT database [22] to benchmark the “health and sustainability” of country-level diets today—or the degree to which diets can satisfy human nutritional needs while respecting an environmental “safe operating space” for food systems [23]. Then, using output from an agricultural projection model, and building on the results initially developed by [24], we produce future projections of the dietary quality of country-level food availability to 2050 under different scenarios of global economic and demographic trends to: a) explore whether diets become more (or less) healthy and sustainable over time under uncertain socioeconomic futures, and b) explore the effect of traded agricultural products on dietary health and sustainability.

## Understanding the evolution of dietary quality

Unhealthy diets are one of the largest risk factors driving the global disease burden [25]. As there is no universally agreed-upon notion of what constitutes a healthy diet, there is also no clear agreement on what constitutes best-practice metrics to measure dietary quality from a holistic and global perspective. A variety of approaches exist to measure dietary quality (for a recent overview, see [26], but many of these are not applicable at the global level. The challenge is compounded when looking at food system sustainability, as the measurements of different aspects of human nutrition at the global scale often do not address the goals of dietary health and environmental sustainability in tandem [27–29].

The transition from a food security focus to a food system emphasis in food policy includes the recognition of a need to develop and track quantitative indicators of multiple facets of global food systems functions. In order to ensure that progress is being made towards ambitious system-level goals, consistent and comparable measures are required to understand whether food systems are functioning in a healthy, equitable, and sustainable manner [30, 31]. Consideration of dietary sustainability in assessments of global diets was a major motivating factor of the EAT-Lancet Commission report on eating within planetary boundaries [23], and the report provided global dietary guidelines to that effect. The EAT-Lancet report motivated a variety of complementary studies [32], and critical discussions [33, 34] as well as comparisons with existing dietary patterns and guidelines [35]. Since then, others have proposed quantitative metrics that operationalize the EAT-Lancet diet as one means to track progress towards sustainable and healthy diets [36].

Extending this line of reasoning, we propose here to examine the role of trade in promoting healthy and sustainable diets on a country-level basis. The EAT-Lancet guidelines have been formulated so as to achieve tandem goals of providing the nutrition necessary for human health at the individual level, while avoiding transgressing an environmental safe operating space at the global level (based on the planetary boundaries framework, see [37, 38]. These are operationalized as maximum and minimum recommended per capita intake for various food groups, which we use to elaborate how trade may drive environmental (un)sustainability and dietary health both today and into the future (2050). To create a measure of dietary health and sustainability, we build upon the approach proposed in [23] to construct metrics which measure dietary quality as a function of 1) average distance from the proposed maximum threshold of EAT-Lancet food group categories and, 2) average distance from a set of proposed minimum thresholds. We then use commodity-level projections of imports and exports from the IMPACT projection model [39] to explore the future effect of trade on our constructed dietary health measures, by comparing a normal trade scenario against a no-trade scenario. We present these results for a globally representative set of countries for four Shared Socioeconomic Pathway scenarios [40, 41].

## Methods

### Constructing dietary health and sustainability measures

Thresholds for EAT-Lancet food group categories were drawn from related studies [42]. Two metrics were constructed to measure the percent overshoot (of maximum thresholds) and percent undershoot (of minimum thresholds) of key dietary elements. The EAT-Lancet dietary thresholds outlined in [23] are measured in grams per capita per day terms (see Table 1).

**Table 1:** Minimum and maximum recommended dietary intake of 9 food categories—Based on EAT-Lancet Dietary Guidelines and [42].

EAT/Lancet Food Group Category	Minimum Intake Threshold (g/cap/day) Based on Hanley-Cook et al. (2020)	Maximum Intake Threshold (g/cap/day) Based on Willet et al. (2019)
***Grains***	32% of Total Dietary Intake	60% of Total Dietary Intake
***Meats (Excluding Fish)***	56	111
***Tubers***	50	100
***Vegetables***	200	600
***Fruit***	100	300
***Dairy***	250	500
***Legumes***, ***Nuts***, ***and Seeds***	100	250
***Added Fats and Oils***	20	92
***Sugars***	0	31

**Fig 1:**
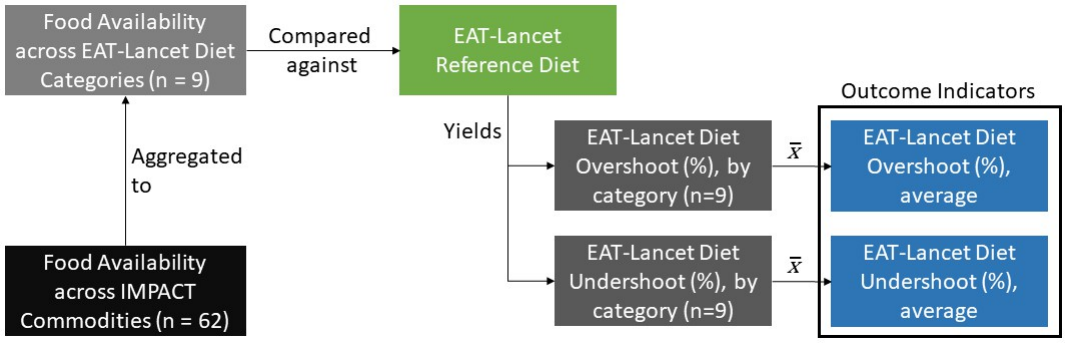
Aggregation and computation method for dietary quality metrics—Overshoot of EAT-lancet dietary guidelines and undershoot of EAT-lancet dietary guidelines.

In determining overall dietary overshoot/undershoot at the country level, the percent overshoot/undershoot of each food group category is averaged by unweighted mean (as the EAT-Lancet metric is threshold-based and thus already contains implicit weights). This yields the *average percent departure from EAT-Lancet dietary thresholds*, both in dietary overshoot and dietary undershoot terms. Fig 1 overviews the method employed for constructing the independent overshoot and undershoot metrics.

### Data

To compute these metrics, data on production, imports, exports, and food availability was first obtained from FAOSTAT [22], covering 116 commodity classes for a set of 182 countries. Commodity classes from the FAOSTAT were assigned to EAT-Lancet food group categories and aggregated. Then, data was taken from the IMPACT agricultural partial equilibrium model [24], from the years 2005 (the model’s base run year) to 2050 –see below for details. In our case, data series for production, import, export, and food availability statistics for 62 modeled agricultural commodity types were aggregated into EAT-Lancet food group categories (Table 1) for 157 countries across all projection years (2005–2050). Conversion factors from the GENuS database [43] for excluding non-edible food portions were then applied to both FAOSTAT and IMPACT data sources and region-specific consumption loss factors are applied to FAOSTAT and IMPACT data sources from Gustavsson et al. [44].

### Extending quantitative foresight model results

For the foresight component of the analysis, we use annual projection data from the International Model for Policy Analysis of Agricultural Commodities and Trade (IMPACT) version 3.0 [39]. IMPACT is a widely-used [e.g. 19, 45–47] multimarket, partial equilibrium model of the agricultural system which incorporates a path solving method for production, demand, prices, trade, and harvested area under conditions of climate change. The model architecture incorporates modules of climate systems, water systems, crop production systems, value chains, and land use. The IMPACT model is used, maintained, and extended by scientists across the CGIAR and the Global Futures and Strategic Foresight (GFSF) program [47]. Here, we use production, demand, and trade output data from IMPACT model runs for a set of four of the Shared Socioeconomic Pathways scenarios (“SSPs”; [40, 41]. Full documentation of the IMPACT model’s architecture and run methodology can be found in [39].

### Scenarios and key assumptions

While the EAT-Lancet diet has been used for scenario analysis in the original EAT commission report [23], we focus here on framing broad future uncertainties related to the global food system that are not explored in the original report. Two major structural drivers of food systems on the demand side are population growth and income growth [29].

We use the Shared Socioeconomic Pathways framework to drive projection of dietary quality under four (SSPs 2–5) structurally different socioeconomic futures. These scenarios provide different trajectories of population and GDP growth which are exogenously imposed on the IMPACT modeling system to introduce demand-side changes. GDP and Population growth rate values are adapted from the SSP public database [48] and used as exogenous inputs in the IMPACT system. The resultant differences in patterns of income and population (Table 2) drive changes to commodity market outcomes (demand, imports, exports) in the model, which results in different trade patterns, and ultimately, dietary availability. Commodity preference patterns within the model are conditioned by demand elasticities which change over the course of the model run horizon to reflect Engel’s Law [49–51].

**Table 2:** Global average GDP per capita and population values for the IMPACT model base year (2005) and four select SSP scenarios. Based on data from IIASA public SSP database (https://tntcat.iiasa.ac.at/SspDb/).

	GDP per Capita (000$)	Population (Billions)
**2005**	8.9	6.5
**SSP2 (2050)**	25.2	9.2
**SSP3 (2050)**	17.9	10
**SSP4 (2050)**	24.1	9.1
**SSP5 (2050)**	42.3	8.6

These SSPs are compatible with climate change assumptions of a 1C-2C increase in global mean surface temperature over preindustrial levels by 2050 (RCP 8.5 scenario—see [52, 53]. This assumption is in-line with analysis which supports a 1C-2C increase in global mean temperature as a logical baseline or “business as usual” concentration pathway [54, 55]. The foresight model results then allow us to calculate future values for the dietary overshoot/undershoot indices, based both on what countries produce themselves and how trade differentially impacts each national food system.

## Results

The analysis includes 142 countries and 16 aggregated regions, covering most of the planet’s populated area. We begin by establishing projections for each SSP scenario of dietary overshoot and undershoot along each food group category. We then look at projections of traded dietary volume to 2050 to get a sense of how much more trade-dependent diets are expected to become on a regional basis. We then evaluate the current and future role of trade in supplying healthy and sustainable diets vis-à-vis alleviating dietary overshoot and undershoot independently.

### Current distribution of global dietary health and sustainability

**Fig 2:**
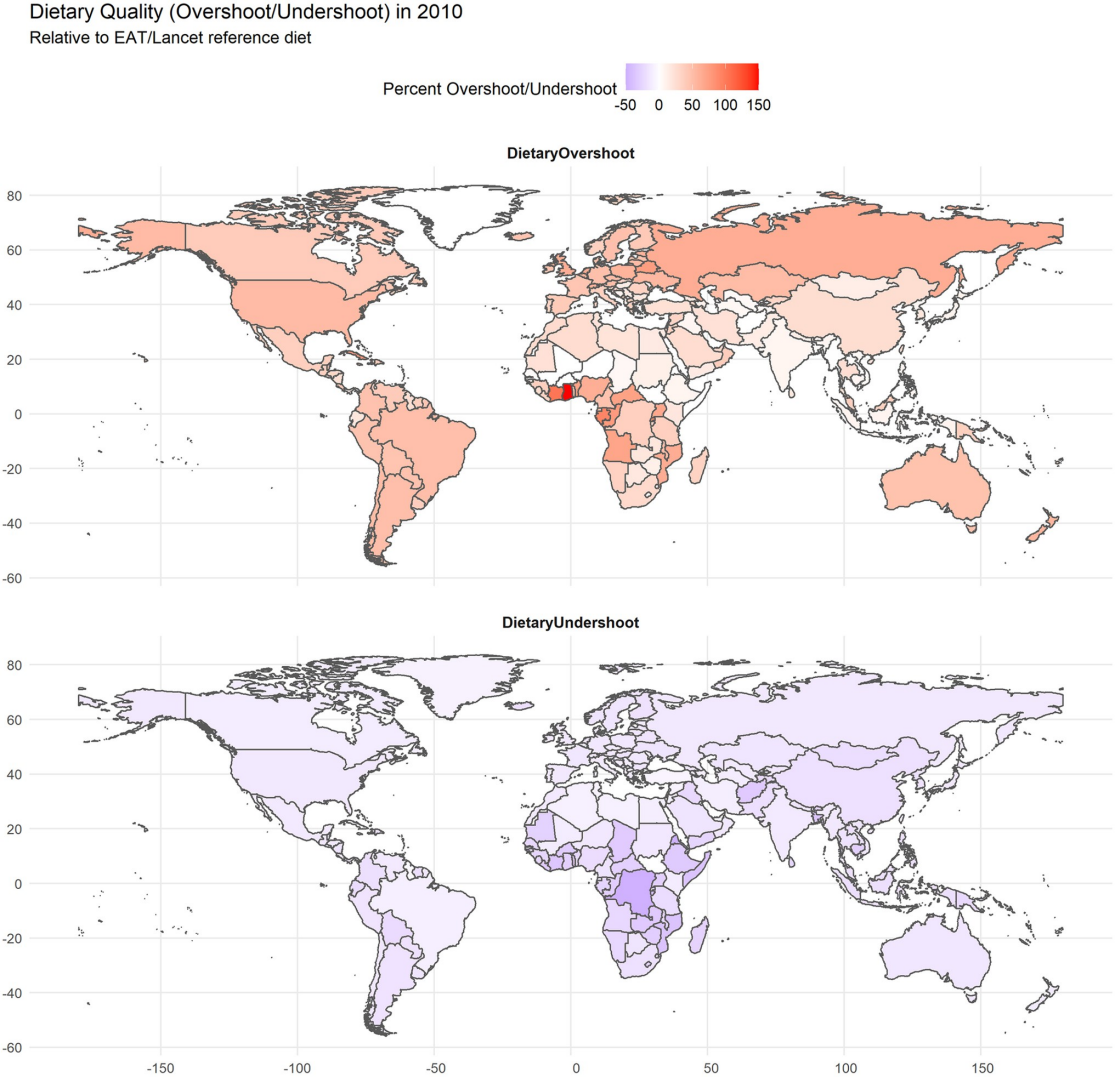
Percent overshoot and undershoot of EAT-Lancet recommended dietary guidelines for a global set of countries in the year 2010, based on FAO data. Where no data exists for country-level diets, values from the IMPACT model run for SSP2 are used. Values closer to zero indicate more “ideal” dietary outcomes with respect to EAT-Lancet guidelines. Source: Author’s computation. Shapefiles for map generation sourced from the NaturalEarth project (naturalearthdata.com).

Fig 2 displays country-level total dietary overshoot (red) and undershoot (blue) values relative to the EAT-Lancet dietary thresholds for the year 2010. Geographically, countries which have lower dietary overshoot outcomes are clustered in the Middle East/North African, East Asia Pacific, and South Asian regions, while countries with the lowest dietary undershoot outcomes are high-income countries and several countries in South America, as well as the Middle East/North Africa region. In contrast, negative outcomes for both dietary overshoot and undershoot are concentrated in the Sub-Saharan African region, confirming that several countries in that region are struggling with a “double burden” dynamic regarding dietary quality.

### Dietary health and sustainability projections

**Fig 3:**
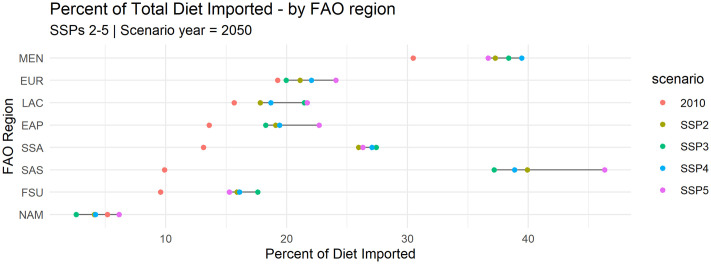
2050 projected average “distance” (overshoot or undershoot) from ideal diet (zero = ideal). Regional averages are a population-weighted average of country-level overshoot/undershoot as projected by the IMPACT model. Regions as follows: SSA = Sub-Saharan Africa, SAS = South Asian States, NAM = North America, MEN = Middle East/North Africa, LAC = Latin America and Caribbean, FSU = Former Soviet Union, EUR = Europe, EAP = East Asia Pacific.

Using the results from Fig 2 as a point of departure, we then look at projecting dietary health and sustainability on a country-level basis with the IMPACT model (Fig 3). For this, dietary overshoot and undershoot are organized by FAO regions for the year 2050 for scenarios SSPs 2–5. The analysis shows that for most regions, the largest dietary overshoot is projected to be under SSP5 –for some regions like the South Asian region, dietary overshoot under SSP5 is more than double the level of expected overshoot under SSP3 for instance, and more than four times the level of overshoot in 2010.

Fig 3 also indicates that changes in dietary undershoot are projected to be less pronounced over time than changes in overshoot. Except for sub-Saharan Africa (SSA), South Asia (SAS), and the East Asia/Pacific region (EAP), progress in reducing dietary undershoot is expected to be quite limited. Furthermore, where advances are expected to be made in reducing dietary undershoot (SSA, SAS), the degree of progress is, to some extent, scenario-dependent. Under an SSP4 future (high international inequality) progress in reducing undershoot for sub-Saharan Africa (SSA) is less than half that of progress expected under SSP5 (high growth, low population).

In regions where the situation of dietary undershoot may improve under some socioeconomic scenario paths, progress is however partially offset by a substantial increase in dietary overshoot. Countries in the Middle East/North African (MEN), North American (NAM), Former Soviet (FSU), and European regions (EUR) for example see dietary undershoot that is functionally the same or potentially worse than dietary undershoot in 2010, while progress in reducing dietary undershoot in Latin American and Caribbean regions (LAC) is expected to occur across a similarly narrow band of possible outcomes.

The SSP5 scenario is projected to drive the most progress in reducing dietary undershoot across regions but is also projected to drive a significant increase in dietary overshoot—almost 60% higher on average than the next highest scenario (SSP2). This implies that the increase in average purchasing power may be effective in some regions (SSA and SAS mainly, and to a limited degree in LAC, and EAP) for reducing dietary undershoot, but at the cost of driving overconsumption of other food commodities. In other regions (EUR, FSU, MEN, NAM), increased purchasing power may drive overconsumption with regards to the EAT-Lancet diet, but with little to no reduction in underconsumption.

### The role of trade in current and future diets

**Fig 4:**
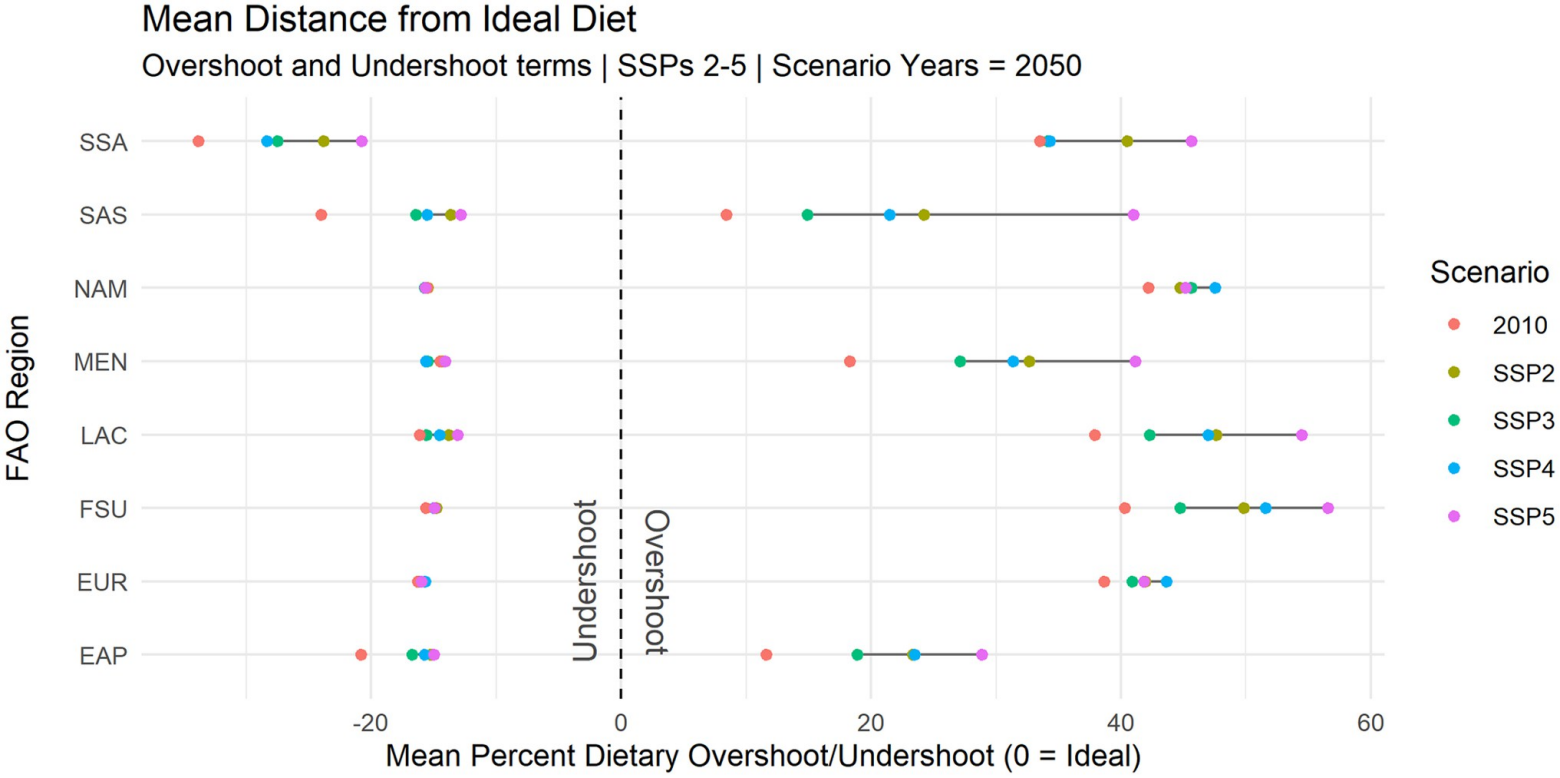
2050 projected percent of total diet that is satisfied through imported goods. Region-level values are population-weighted country-level averages of the portion of average diet imported. Regions as follows: SSA = Sub-Saharan Africa, SAS = South Asian States, NAM = North America, MEN = Middle East/North Africa, LAC = Latin America and Caribbean, FSU = Former Soviet Union, EUR = Europe, EAP = East Asia Pacific.

The effect of trade on healthy and sustainable diets is in some sense bounded by the portion of the average diet that is derived or expected to be derived (for future years) from traded goods. Fig 4 tracks projections of the percent of total diet derived from imported foods across different geographic regions. While in 2010 traded agricultural commodities account for an average of 14% of global diets, projections from IMPACT suggest that traded commodities will account for 26–29% of global diets by 2050, with significant regional variations. The percent of dietary energy derived from traded goods is also somewhat sensitive to socioeconomic conditions. For most regions, scenarios with higher per capita income assumptions (SSP5) drive a higher percent of dietary energy from trade, save for Sub-Saharan Africa (SSA) and former Soviet Union states (FSU). SSA projections from IMPACT imply that the region may become significantly more import-dependent with respect to average diets than it has been historically, and that this pattern is persistent across possible socioeconomic scenarios. This contrasts with the South Asian region (SAS) for example, where an even larger increase in trade dependency of diets is projected, but with an almost 10 percentage point variation depending on socioeconomic circumstance.

As agricultural trade is expected to increase in importance for the average diet out to mid-century, rather than decrease, it is prudent to try and understand whether countries with larger increases in trade (a key driver of food systems—see e.g. [29] are expected to have food environments that are more or less enabling for human and planetary health. Table 3 presents the cross-country correlations between the change in level of per capita imports between 2010 and 2050 with the change in both measures of EAT-Lancet dietary quality (overshoot and undershoot) for the same period. Following the analytical approach from [15], if imported commodities were linked to more healthy and sustainable diets, we would expect that increases in imports per capita should correlate with decreases in both dietary undershoot and overshoot in 2050 (i.e., a negative relationship between the two variables). We find **1)** weak but significant negative relationships between change in imports per capita and dietary undershoot across all scenarios, suggesting that regions with larger change in import volume relative to 2010 are relatively more successful at reducing dietary undershoot regardless of global socioeconomic patterns; and **2)** weak but significant positive associations between dietary overshoot and change in imports per capita across all scenarios except SSP3, suggesting that regions with larger change in imports per capita are relatively less successful at containing dietary overshoot except in a scenario where socioeconomic outcomes are fairly stagnant (SSP3).

**Table 3:** Correlation between the change in the level of agricultural imports per capita and change in the dietary overshoot and dietary undershoot metrics between 2010–2050 for SSPs 2–5. Statistically significant relationships are shown in bold.

Diet Measure	Scenario	r	p
**Overshoot**	SSP2	**0.34**	<0.001***
	SSP3	0.14	0.087
	SSP4	**0.16**	0.046*
	SSP5	**0.37**	<0.001***
**Undershoot**	SSP2	**-0.32**	<0.001***
	SSP3	**-0.24**	0.002**
	SSP4	**-0.2**	0.013*
	SSP5	**-0.28**	<0.001***

Given that many of the regions expected experience dietary undershoot are made up of lower-income countries, we additionally grouped countries by income quartile and reevaluated the relationship between change in imports and dietary quality measures. After accounting for level of income in this way, we observe different patterns in the relationship between overshoot and change in imported foods (Table 4). Upper middle-income regions display a moderately positive relationship between change in imports and change in overshoot across all scenarios, while low-income regions display a moderately positive relationship between change in imports and change in overshoot under SSP2 and SSP5. This suggests that under low-growth futures for low-income countries (SSP3, SSP4), increases in imports are not expected to be strongly associated with dietary overshoot, and are relatively smaller overall.

**Table 4:** Cross-country correlations tracking the change in country-level overshoot (arithmetic mean of food group category-level overshoot) between 2010 and 2050 and the change in imports per capita between the years 2010 and 2050. Aggregation by income is via global income quartiles, and are roughly analogous to World Bank income groupings. Income quartiles groupings are static over time (i.e., the income designation of a given country in 2010 persists through 2050). Correlation coefficients are calculated at the income group level.

Diet Measure	Scenario	Income Level	r	p
**Overshoot**	SSP2	High Income	0.11	0.498
		Low Income	**0.47**	0.003**
		Lower Mid Income	0.23	0.156
		Upper Mid Income	**0.48**	0.002**
	SSP3	High Income	-0.01	0.968
		Low Income	0.14	0.408
		Lower Mid Income	0.08	0.624
		Upper Mid Income	**0.43**	0.006**
	SSP4	High Income	0.11	0.489
		Low Income	0.01	0.554
		Lower Mid Income	0.11	0.492
		Upper Mid Income	**0.41**	0.01*
	SSP5	High Income	0.13	0.423
		Low Income	**0.50**	**0.001**
		Lower Mid Income	0.26	0.105
		Upper Mid Income	0.28	0.082

Table 5 shows the relationships between change in imports and change in dietary undershoot by income level. Here, only low-income countries show moderate negative correlations across all scenarios. This indicates that increases in import volume per capita over time are projected to be associated with better dietary undershoot outcomes only for low-income countries but have little impact on closing the underconsumption gap elsewhere.

**Table 5:** Cross-country correlations tracking the change in country-level undershoot (arithmetic mean of food group category-level overshoot) between 2010 and 2050 and the change in imports per capita between the years 2010 and 2050. Income regions are constructed via global income quartiles, and are roughly analogous to world bank income groupings. Income quartiles groupings are static over time (i.e., the income designation of a given country in 2010 persists through 2050). Correlation coefficients are calculated at the income group level.

Diet Measure	Scenario	Income Level	r	p
**Undershoot**	SSP2	High Income	0.11	0.508
		Low Income	**-0.47**	0.002**
		Lower Mid Income	-0.13	0.443
		Upper Mid Income	-0.07	0.691
	SSP3	High Income	0.11	0.488
		Low Income	**-0.44**	0.006**
		Lower Mid Income	-0.15	0.375
		Upper Mid Income	0.1	0.533
	SSP4	High Income	0.08	0.626
		Low Income	**-0.41**	0.009**
		Lower Mid Income	-0.18	0.283
		Upper Mid Income	0.03	0.87
	SSP5	High Income	0.03	0.882
		Low Income	**-0.45**	0.004**
		Lower Mid Income	-0.045	0.785
		Upper Mid Income	-0.14	0.406

**Fig 5:**
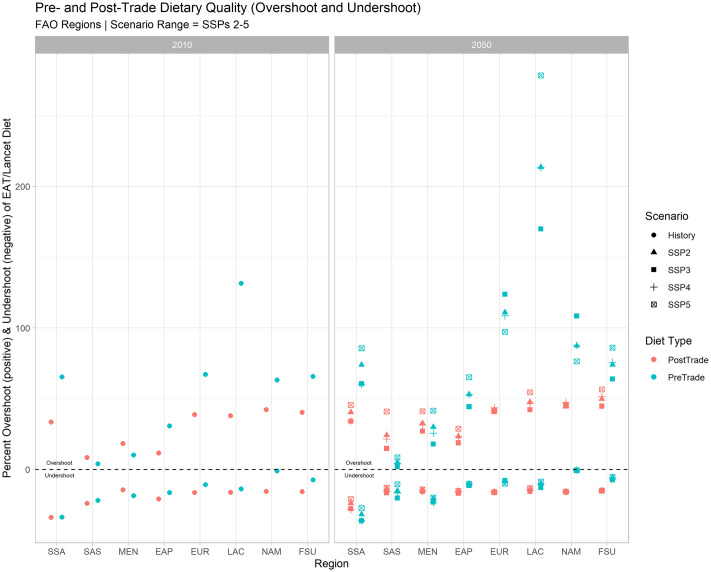
Regional comparison of “pre-trade” (production-only) average diets and actual average diets in overshoot and undershoot terms. Regional-level metrics are constructed as a population-weighted mean of average country-level dietary undershoot or overshoot.

If we expect agricultural trade to affect dietary quality in a mostly negative manner due to the potential for increased overshoot, we might also ask whether less trade would provide for better dietary outcomes. To answer this question we estimate a hypothetical “no-trade” diet (see, e.g. [56], which consists of all of the agricultural goods that a given country produces, minus goods destined for non-food processing (biofuel stock, etc.). We then estimate dietary overshoot and undershoot metrics for the pre-trade diet and compare the values against projected diets *including* food trade (i.e., country-level food availability), to assess the degree of benefit derived from agricultural trade. These results are displayed in Fig 5.

From this comparison, we can see that in 2010 (left panel), dietary undershoot conditions are slightly improved by consuming a “pre-trade” diet across most regions. In the North American region (NAM) for example, the dietary undershoot gap is closed nearly completely by eating only what countries in this region produce. This is reversed for the MEN region, and dietary undershoot in the Sub-Saharan African region (SSA) is roughly comparable both before and after trade. On the dietary overshoot side however, most countries experience a large benefit from global trade in terms of reduction of dietary overshoot. Latin American diets, for example, see a nearly 100 percentage point closure of the dietary overshoot gap achieved via agricultural trade in 2010. The MEN and South Asian (SAS) regions, however, see dietary overshoot increased through international food trade. South Asian states are the only region that see both dietary undershoot and dietary overshoot performance become worse after accounting for the effect of trade.

We then project the effect of trade on diets to 2050 under the four SSPs used for this study (right panel). Here we observe that South Asian (SAS) states are still projected to see dietary health and sustainability reduced by international food trade across most scenarios (excluding SSPs 2–3). By contrast, Sub-Saharan Africa (SSA) is projected to benefit from international food trade overall (reducing both overshoot and undershoot). For most other regions, reductions in dietary undershoot are still expected to be at least partially offset by international food trade, with the dietary outcomes on the undershoot side remaining more or less the same as in 2010 across most scenarios. On the overshoot side however, the degree to which international food trade benefits dietary quality is expected to increase under most scenarios and most regions. Latin American countries (LAC), for instance, are expected to see their dietary overshoot decrease by over 200 percentage points after accounting for traded foods under a high growth—low population future (SSP5).

**Fig 6:**
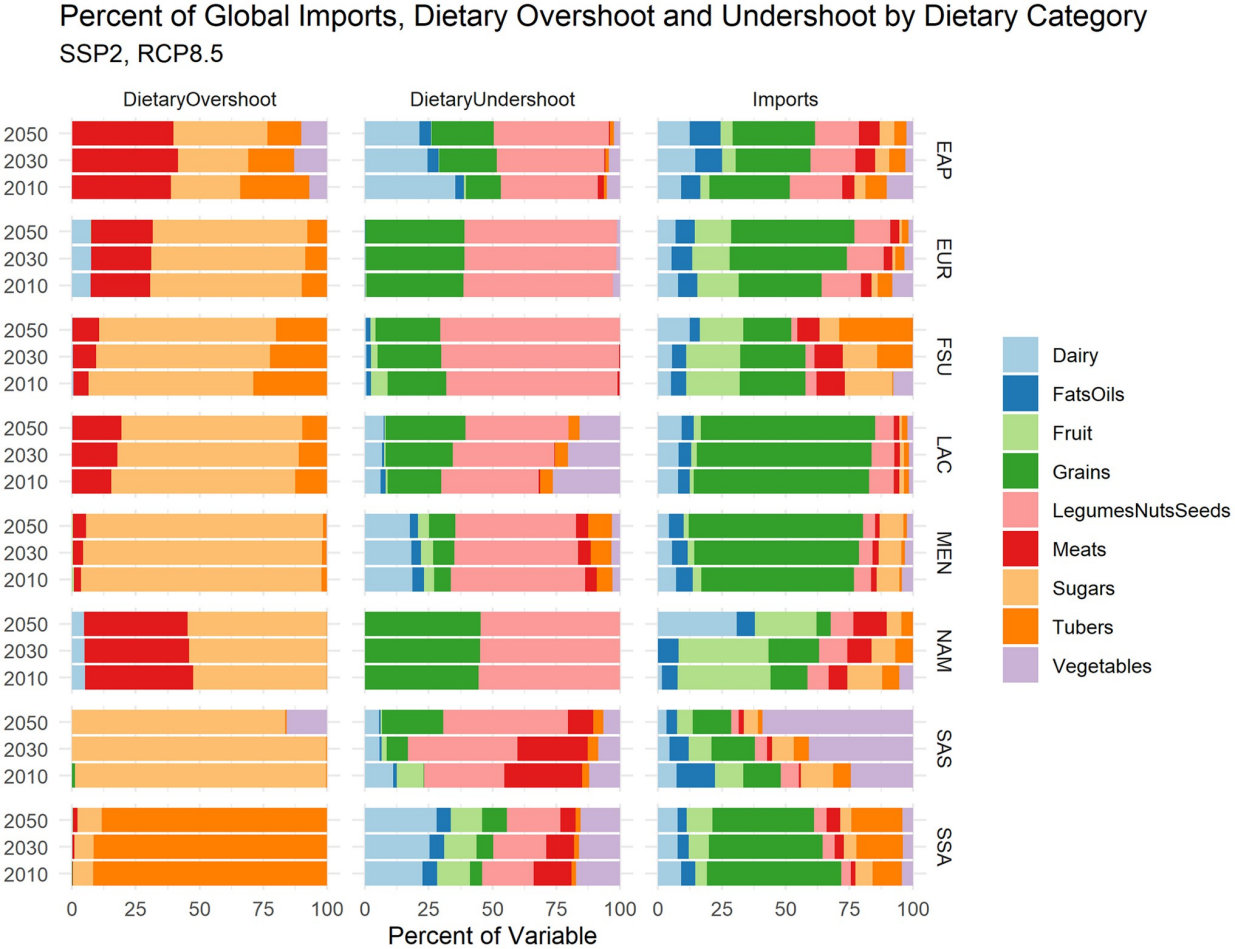
Regional percentages of dietary undershoot and overshoot represented by EAT-Lancet dietary category. Regional-level metrics are constructed as a population-weighted mean of country-level overshoot (left), undershoot (middle), and import volume (right). As the percent decomposition is a relative metric, the absolute size of any dietary category is not comparable across regions, but should be observed against other dietary categories across time for a given region.

While traded commodities are found to benefit most global regions (on average) from our dietary analysis—there are several countries for which trade is expected to continue to affect negatively dietary health and sustainability on both overshoot and undershoot sides. In order to understand why a certain country or region may or may not benefit in dietary terms from agricultural trade, we then looked into the food group categories that are contributing to dietary overshoot and undershoot at the regional level in Fig 3, and explored where trade patterns might be misaligned with more ideal dietary outcomes in this regard (Fig 6). Where a high degree of overshoot, for example, is projected within a certain food group category for a given year AND a high volume of the food group category is imported (for a particular region) relative to other imports, we might expect that this points to a situation where trade outcomes are misaligned with dietary outcomes.

Fig 6 breaks out dietary overshoot, dietary undershoot, and import volume for the EAT-Lancet food group categories that we used to construct the dietary quality metrics. These food group categories are measured by variables on a proportional basis (i.e., percent of total imports, percent of total dietary overshoot, etc.). The results are displayed for the years 2010, 2030, and 2050.

Fig 6 shows that dietary overshoot across regions is dominated essentially by sugars (save for SSA) and meats. For most regions, the proportion of sugars imported (rightmost column) relative to other food group categories is expected to decrease slightly over time, but proportional overshoot burden is expected to increase or remain the same. On the undershoot side, most regions’ dietary undershoot is dominated by Legumes, Nuts, and Seeds (LNS)–and this undershoot is projected to increase in most regions to 2050. However, many regions import very little LNS compared to other food group categories, and shares of imported LNS are expected to decline compared to other food group categories across many regions.

## Discussion

In this study, we sought to untangle the expected future relationship between international food trade and healthy and sustainable diets for a globally-representative set of countries, leveraging established projection models and socioeconomic scenarios [41, 57]. Understanding better the role of trade in promoting or impeding healthy diets and environmental sustainability across different countries is fundamental for ensuring that the global sustainability agenda is aligned with reduction of the triple burden of malnutrition conjointly [1, 58–60]. Achieving this policy confluence, especially in light of calls to expand food trade to address e.g. vulnerabilities created by climate change [14] will be one of the major challenges of the next few decades.

### Broad trends around diets and trade

Our analysis shows that under projections of international trade environments that are fundamentally price-driven (i.e., following the logic of the IMPACT model), dietary health and sustainability benefits/losses from trade are projected to accrue to different sets of countries in different ways. Problems of overconsumption are expected under this projection framework to continue to be a major issue for dietary health and sustainability for large parts of the world out to mid-century. Under a high-income per capita scenario (SSP5) dietary overshoot is projected to be significantly worse in several regions compared to other scenarios. Low-income countries, which tend to struggle relatively more with underconsumption in dietary quality terms, are projected to see positive returns from agricultural trade in reducing dietary undershoot. However, under either “business-as-usual” (SSP2) or high-growth (SSP5) scenarios, undershoot reduction is expected to occur alongside increased dietary overshoot for those low-income countries. This outcome nuances in the utility of increased purchasing power for enabling better food systems outcomes in low-income countries, where, under demand-driven trade environments absent substantial policy intervention, small advances in reduction of underconsumption are expected to be granted at the expense of large advances in overconsumption with respect to human health and environmental wellbeing.

Because overshoot and undershoot of EAT-Lancet dietary guidelines are projected to ‘co-exist’ for most regions under future scenarios, a “double-burden” dynamic of over- and under consumption with respect to the EAT-Lancet dietary guidelines is expected. For upper-middle income countries especially, and low-income countries to a lesser degree, global agricultural trade may exacerbate a dietary double-burden dynamic under some scenarios (SSP2, SSP3, SSP4 for upper-middle income, SSP2, SSP5 for low-income—Tables 4 and 5). While this dynamic does not persist clearly across other income groups, some geographic heterogeneity is observed (Fig 5). This can be most clearly seen in the South Asia region, where a “no-trade” diet is expected to continue to outperform actual diets including traded goods in some scenarios.

### Implications for food trade policies

Looking beyond overall diet to dietary components (Fig 6), we can observe that the composition of traded goods over time are not necessarily aligned with improved dietary quality over the course of business-as-usual scenario conditions (SSP2). For example, some regions (EAP, NAM) might be expected to expand imports of sugars or meats even while the per capita availability of these products continues to be much higher than that recommended by the EAT-Lancet diet. Other regions (LAC, FSU, SAS) see availability of legumes, nuts, and seeds much lower than what would be needed to satisfy the EAT-Lancet diet, at the same time as the import share of these commodities is expected to fall over time. Levels of legumes, nuts, and seeds in diets across regions into the future are generally consistently lower than the EAT-Lancet dietary guidelines, and this is important to consider in tandem with concerns that the production environments necessary to satisfy these levels may exacerbate water stress [34].

Our category-based measurements of dietary health and sustainability nuances the narratives that trade advances malnutrition reduction in certain regions of the world [61] and draws attention to a more heterogenous role of trade in securing healthy and sustainable diets [62–64]. Countries which are expected to not experience dietary benefit from trade on either the overshoot or undershoot side of the equation may actually see dietary benefits from more limited engagement with global agricultural markets, or reformed trade policies that promote nutritional goals [63, 65–69].

For global food trade, this means that the trade system will need to adapt to continue to provide solutions to issues of undernutrition in some areas (but with a focus on more flexibly supplying different types of foods necessary for healthy diets) as well as to limit the oversupply of certain types of foods (sugars, meats, etc.–[70, 71]. This is an immense challenge that extends beyond allocating goods more efficiently to in-demand markets, where in some cases more efficient allocation of the wrong types of goods will drive poorer dietary quality and sustainability outcomes.

### Opportunities for further analysis

While this study generates new important insights into the expected role of trade in contributing to healthy and sustainable diets into the future, it is also limited in several ways. First, the study uses measures of food availability from FAOSTAT both to generate the baseline global dietary conditions, as well as to calibrate the IMPACT model projections. While this is an invaluable source of cross-country normalized agricultural statistics, it has been noted previously to potentially overestimate consumption of certain food groups while underestimating consumption of others [72]. Thus, the results and implications of our study are predicated upon the accuracy of the FAO statistics.

Second, while IMPACT is a useful tool for dietary analysis due to the high number of unique agricultural commodities it represents relative to other global agricultural modeling platforms, it still falls short of representing the full suite of commodities represented in FAOSTAT. IMPACT also provides a simplified representation of commodity trade due to the “pooled” markets structure of the model (individual countries trade with a single global market), so our results here are thus best interpreted as following from a “baseline” trade future, which is largely price-driven and tends toward country-level specialization on the production-side. Against this background, it is important to keep in mind that political economic considerations [1] will continue to shape future trade systems in unexpected ways. Explicit analysis of shifts in bilateral trade were not possible for this study, but remain an important potential avenue for future research, especially relating to, e.g., the potential impact of trade “chokepoints” on achieving healthy diets [73].

There are also some important considerations for these results stemming directly from the EAT-Lancet diet’s formulation. For instance, the significant dietary overshoot in the Sub-Saharan African region is mostly associated with a large portion of dietary energy coming from roots and tuberous foods—for which the EAT-Lancet dietary guidelines set a relatively low maximum threshold (100 g/cap/day) citing high glycemic load associated with the consumption of tubers and starchy vegetables [23, 74]. Processing of grains into highly refined foods such as white bread makes grain-derived foods comparable to tubers in terms of glycemic load [75], and because our study does not consider food preparation method, it is almost certainly the case that the health benefits from grains versus tubers are skewed here in favor of grains. Future avenues for study into cross-country trends in dietary health using EAT-Lancet should be sensitive to the role of food processing in driving overshoot or undershoot of different category-based dietary thresholds.

## Conclusion

The world today faces challenges on two distinct fronts of dietary health and sustainability—with countries both undershooting (falling short) and overshooting (exceeding) many dietary health and sustainability goals and limits. The dietary metrics constructed in this paper present a way to track progress towards, or movement away from, “ideal” dietary and sustainability states, and the results make clear that managing the balance between improving dietary undershoot and limiting dietary overshoot will continue to be a major challenge across countries and regions globally in the future. The results further show that trade, though expected to provide benefits for dietary health and sustainability to many countries, is also projected to exacerbate dietary harms in others under business-as-usual trade conditions. We find this to be true to varying degrees when accounting for potential socioeconomic uncertainty under the SSP framework, evidencing that socioeconomic patterns of development will have implications for both the impact of trade on dietary outcomes, and dietary outcomes themselves. In general, we find that scenarios with higher growth conditions imply far greater dietary overshoot potential across a number of regions (Fig 3) while sometimes making only very limited progress towards reducing dietary undershoot. Under such scenarios, new avenues for managing food demand and the dietary environment may need to be explored given the dearth of demand-side policy approaches combining human and planetary health concerns [76]. We also find the role of trade to be a possible driver of both reduction in dietary overshoot in low-income countries (across all scenarios, see Table 5) and expansion of dietary overshoot in low- and upper-middle income countries (scenario dependent, see Table 4). Sugars and Meat products make up the vast majority of the current and expected future dietary overshoot burden across countries (Fig 6), in line with the observed global transition to a more “western diet” [77–79]. As the imported volume of these goods are expected to increase across many regions, more careful management of the trade environment may be a potential lever for minimizing and managing deleterious effects on dietary health and sustainability stemming from these food groups specifically. Legumes, Nuts, and Seeds, along with Grains, present the largest dietary gaps across regions out to 2050, though these results must be qualified against criticisms of high volumes of Legumes/Nuts/Seeds in the EAT-Lancet diet, and the sensitivity of the dietary thresholds for Grains to overall dietary volume (as target thresholds for Grains are set at 32–60% of total dietary intake). In general, the commodity breakdown of dietary gaps (undershoot) is more varied across regional contexts than the transgression of dietary and sustainability boundaries (overshoot), meaning that filling remaining gaps in sustainable and healthy diets presents a more complex and context-specific challenge than managing overshoot, which may require more targeted engagement with a smaller set of food group sectors.

In sum, while there is much room for trade policy to be re-aligned and create healthy and sustainable dietary incentives, it cannot be relied upon as a tool to address dietary health in isolation. Rather, other policy interventions on both the production and demand side of diets will need to be undertaken in conjunction with trade policy in order to address global dietary challenges, especially those related to overconsumption of food groups that are expected to be widely over-available (meats, sugars) or under-available (legumes, nuts, and seeds).
